# Sampling methods in Clinical Research; an Educational Review

**Published:** 2017-01-14

**Authors:** Mohamed Elfil, Ahmed Negida

**Affiliations:** 1 Faculty of Medicine, Alexandria University, Egypt.; 2 Faculty of Medicine, Zagazig University, Egypt.

**Keywords:** Research design, sampling studies, evidence-based medicine, population surveillance, education

## Abstract

Clinical research usually involves patients with a certain disease or a condition. The generalizability of clinical research findings is based on multiple factors related to the internal and external validity of the research methods. The main methodological issue that influences the generalizability of clinical research findings is the sampling method. In this educational article, we are explaining the different sampling methods in clinical research.

## Introduction

In clinical research, we define the population as a group of people who share a common character or a condition, usually the disease. If we are conducting a study on patients with ischemic stroke, it will be difficult to include the whole population of ischemic stroke all over the world. It is difficult to locate the whole population everywhere and to have access to all the population. Therefore, the practical approach in clinical research is to include a part of this population, called “sample population”. The whole population is sometimes called “target population” while the sample population is called “study population. When doing a research study, we should consider the sample to be representative to the target population, as much as possible, with the least possible error and without substitution or incompleteness. The process of selecting a sample population from the target population is called the “sampling method”. 

**Sampling types**

**Figure 1:**
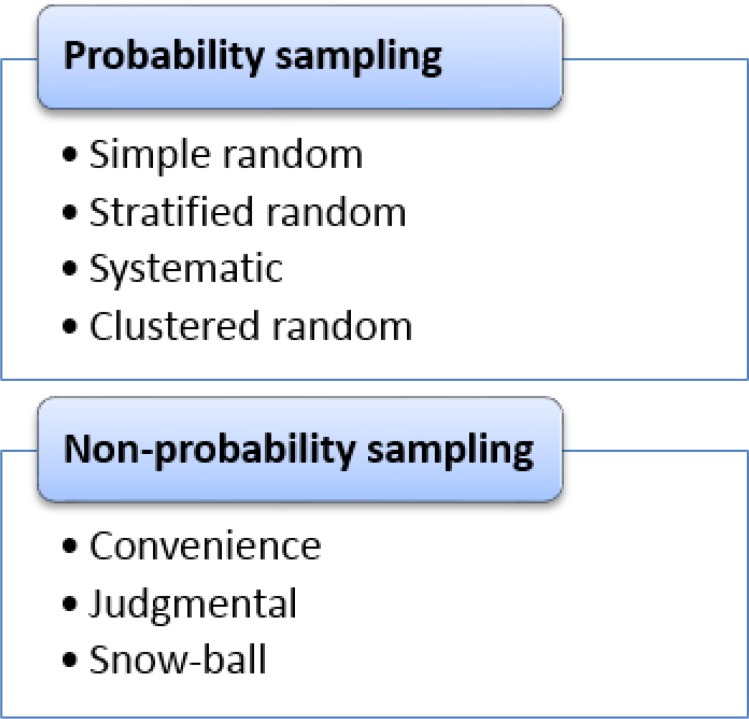
Sampling methods.

There are two major categories of sampling methods (figure 1): 1; probability sampling methods where all subjects in the target population have equal chances to be selected in the sample [1,2] and 2; non-probability sampling methods where the sample population is selected in a non-systematic process that does not guarantee equal chances for each subject in the target population [2,3]. Samples which were selected using probability sampling methods are more representatives of the target population.

**Probability sampling method**

***Simple random sampling***

This method is used when the whole population is accessible and the investigators have a list of all subjects in this target population. The list of all subjects in this population is called the “sampling frame”. From this list, we draw a random sample using lottery method or using a computer generated random list [4].

***Stratified random sampling***

This method is a modification of the simple random sampling therefore, it requires the condition of sampling frame being available, as well. However, in this method, the whole population is divided into homogeneous strata or subgroups according a demographic factor (e.g. gender, age, religion, socio-economic level, education, or diagnosis etc.). Then, the researchers select draw a random sample from the different strata [3,4]. The advantages of this method are: (1) it allows researchers to obtain an effect size from each strata separately, as if it was a different study. Therefore, the between group differences become apparent, and (2) it allows obtaining samples from minority/under-represented populations. If the researchers used the simple random sampling, the minority population will remain underrepresented in the sample, as well. Simply, because the simple random method usually represents the whole target population. In such case, investigators can better use the stratified random sample to obtain adequate samples from all strata in the population.

***Systematic random sampling (Interval sampling)***

In this method, the investigators select subjects to be included in the sample based on a systematic rule, using a fixed interval. For example: If the rule is to include the last patient from every 5 patients. We will include patients with these numbers (5, 10, 15, 20, 25, ...etc.). In some situations, it is not necessary to have the sampling frame if there is a specific hospital or center which the patients are visiting regularly. In this case, the researcher can start randomly and then systemically chooses next patients using a fixed interval [4].

***Cluster sampling (Multistage sampling)***

It is used when creating a sampling frame is nearly impossible due to the large size of the population. In this method, the population is divided by geographic location into clusters. A list of all clusters is made and investigators draw a random number of clusters to be included. Then, they list all individuals within these clusters, and run another turn of random selection to get a final random sample exactly as simple random sampling. This method is called multistage because the selection passed with two stages: firstly, the selection of eligible clusters, then, the selection of sample from individuals of these clusters. An example for this, if we are conducting a research project on primary school students from Iran. It will be very difficult to get a list of all primary school students all over the country. In this case, a list of primary schools is made and the researcher randomly picks up a number of schools, then pick a random sample from the eligible schools [3].

**Non-probability sampling method**

***Convenience sampling***

Although it is a non-probability sampling method, it is the most applicable and widely used method in clinical research. In this method, the investigators enroll subjects according to their availability and accessibility. Therefore, this method is quick, inexpensive, and convenient. It is called convenient sampling as the researcher selects the sample elements according to their convenient accessibility and proximity [3,6]. For example: assume that we will perform a cohort study on Egyptian patients with Hepatitis C (HCV) virus. The convenience sample here will be confined to the accessible population for the research team. Accessible population are HCV patients attending in Zagazig University Hospital and Cairo University Hospitals. Therefore, within the study period, all patients attending these two hospitals and meet the eligibility criteria will be included in this study.

***Judgmental sampling***

In this method, the subjects are selected by the choice of the investigators. The researcher assumes specific characteristics for the sample (e.g. male/female ratio = 2/1) and therefore, they judge the sample to be suitable for representing the population. This method is widely criticized due to the likelihood of bias by investigator judgement [5].

***Snow-ball sampling***

This method is used when the population cannot be located in a specific place and therefore, it is different to access this population. In this method, the investigator asks each subject to give him access to his colleagues from the same population. This situation is common in social science research, for example, if we running a survey on street children, there will be no list with the homeless children and it will be difficult to locate this population in one place e.g. a school/hospital. Here, the investigators will deliver the survey to one child then, ask him to take them to his colleagues or deliver the surveys to them.

## Conflict of interest:

None

## References

[1] J Wretman (2010). Reﬂections on probability vs nonprobability sampling, Ofﬁcial Stat. Honour Daniel Thorburn.

[2] A Shorten, C Moorley Selecting the sample. Evid Based Nurs.

[3] F Gravetter, L Forzano (2012). Selecting Research Participants. Res Methods Behav Sci.

[4] P Sampling, P Guidelines, MS Choices, T Oaks (2012). CHAPTER 5 C hoosing the T ype of.

[5] C Teddlie F Yu Mixed Methods Sampling: A Typology With Examples. J Mix Methods Res.

[6] T Critically A Everyday, Chapter 7 Sampling Techniques Introduction to Sampling Distinguishing Between a Sample and a Population Simple Random Sampling Stratified Random Sampling Convenience Sampling Quota Sampling Sample Size Sampling Error Evaluating Information From Samples , (n.d.).

